# Differences between radiographic and non-radiographic axial spondyloarthritis patients in a Mexican cohort

**DOI:** 10.1038/s41598-024-61001-w

**Published:** 2024-05-06

**Authors:** John Londono, Cesar Pacheco-Tena, Ana Maria Santos, Mario Humberto Cardiel, Gustavo Rodríguez-Salas, Igor Rueda, Sofía Arias-Correal, Cristian Mesa, Mantilla Marta Juliana, Juan Camilo Santacruz, Juan Camilo Rueda, Gilberto Vargas-Alarcón, Rubén Burgos-Vargas

**Affiliations:** 1 Department of Rheumatology and Immunology-Spondyloarthritis Study Group (GESPA), Universidad de La Sabana-Hospital Militar Central, Bogotá, Colombia; 2 PABIOM Laboratory, Faculty of Medicine and Biomedical Sciences, Autonomous University of Chihuahua, 31125 Chihuahua, Mexico; 3 Hospital General “Dr. Miguel Silva”, Instituto Nacional de Ciencias Médicas y Nutrición Salvador Zubiran, McMaster University, Universidad Michoacana de San Nicolas de Hidalgo Instituto de Física y Matemáticas, Morelia, Mexico; 4 Department of Molecular Biology, Instituto Nacional de Cardiología Ignacio Chávez, Mexico City, Mexico; 5 Rheumatology Department, Hospital General de México Eduardo Liceaga, Mexico City, Mexico; 6 Aspirante a Doctorado en Biociencias, Universidad de La Sabana, Chía, Cundinamarca Colombia

**Keywords:** Spondyloarthritis, Ankylosing spondylitis, Rheumatology

## Abstract

To compare the demographic, clinical, and laboratory characteristics, disease onset, and clinical features of radiographic axial Spondyloarthritis (r-axSpA) and non-radiographic axial Spondyloarthritis (nr-axSpA) patients. All patients who attended outpatient spondylarthritis (SpA) clinics at Hospital General de Mexico and the Instituto Nacional de la Nutrición from 1998 to 2005 and met the rheumatologist diagnostic criteria for SpA were selected. Then the SpA patients were classified by European Spondyloarthropathy Study Group criteria (ESSG). We selected SpA patients with axial presentation as axial SpA (axSpA), and they were classified as r-axSpA if they met modified New York (mNY) criteria for sacroiliitis and as nr-axSpA if they did not meet mNY criteria; to compared clinical, demographic, and laboratory test between the subgroups. It included 148 SpA patients; 55 (37.2%) patients had r-axSpA, and 70 (47.3%) had nr-axSpA. The nr-axSpA patients had a lower proportion of males (58.6% vs 78.2%, P < 0.05), lower HLA-B27 frequency (54.3%. vs. 92.7%, P < 0.05), were older at disease onset (21 vs 16 years; P < 0.01) and had a higher frequency of infections at disease onset (9.1% vs 32.9, P < 0.05) than r-axSpA. BASFI (2.9 vs 4.8; P < 0.0001), Dougados functional index (7 vs. 14; P < 0.05), and BASDAI (4.1 vs. 5.2; P < 0.001) were lower in patients with nr-axSpA than r-axSpA, respectively. The factors that most influenced the presentation of r-axSpA were history of uveitis (OR 14, 95% CI 2.3–85), HLA-B27 (OR 7.97, 95% CI, 2.96–122), male sex (OR 6.16, 95% CI, 1.47–25.7), axial enthesopathy count (OR 1.17 95% CI, 1.03–1.33). This study provides insight into the differences between nr-axSpA and r-axSpA in Mexico. Patients with r-axSpA were mainly male, with a younger presentation age, a higher prevalence of HLA-B27, more history of uveitis, fewer episodes of dactylitis, more axial enthesopathy, and higher disease activity than nr-axSpA**.**

## Introduction

Spondyloarthritis (SpA) is a group of chronic inflammatory diseases that share similar clinical and genetic characteristics. SpA is as prevalent as rheumatoid arthritis (RA) in the Latin American population, where SpA prevalence is estimated to range between 0.28 and 0.9%^1,2^. The prevalence of SpA varies around the world, ranging from 0.20% in southeast Asia to 1.61% in the northern Arctic^3^.

The SpA is currently classified by Assessment of SpondyloArthritis international society (ASAS) as axial SpA (axSpA)^4^ and peripheral SpA (pSpA) according to the predominance of clinical presentation^5^, with chronic low back pain in young patient being an important requirement for axSpA, in addition to the presence of the clinical arm (HLA-B27 allele plus two SpA characteristic) or imaging arm (sacroiliitis on imaging plus one SpA characteristic). Otherwise, the pSpA classification includes the presence of dactylitis, enthesitis, or arthritis plus one SpA feature or two other SpA features. The axSpA is subclassified as radiographic (r-axSpA) or non-radiographic (nr-axSpA) according to sacroiliitis by modified New York (mNY) criteria. However, the ASAS classification criteria leave out some patients with the disease because these patients cannot be grouped into a specific disease subtype. Patients with axial symptoms without sacroiliitis in conventional radiography (X-ray) or in magnetic resonance imaging (MRI) of sacroiliac joints (SIJ) with alleles other than HLA-B27 have been reported for more than 40 years^6^.

According to the European Spondyloarthropathy Study Group (ESSG)^7,8^, a considerable proportion of these individuals were previously classified as having undifferentiated forms of SpA (uSpA)^7^. Although a group of nr-axSpA was identified during the development of the ASAS axial criteria, they were not the focus of the classification and did not receive acknowledgment as independent category or proper characterization. However, since 2013, clinical studies have been conducted to assess different treatments in this subgroup of patients^9^. Now, collaborative work is being conducted to improve and validate the ASAS classification, which will help to enhance the detection of more patients (CLassification of Axial Spondyloarthritis Inception Cohort (CLASSIC).

The modifications made to the classification of the disease are the result of a more comprehensive understanding of the disease and a greater emphasis on previously under-studied subgroups, such as nr-axSpA. There are some hypotheses about nr-axSpA being put forward, including the possibility of an evolutionary stage leading to ankylosing spondylitis (AS), a frustrated form of AS, or a different disease subtype that has not been fully recognized and described^10^. Certain factors are associated with the presentation of nr-axSpA or r-axspA^11^. There is little evidence in Latin America on nr-axSpA patients, more data need to be published in this region, where patients with nr-axSpA and r-axSpA are characterized and compared according to the current classification criteria.

This study aimed to characterize and compare a Mexican SpA cohort with nr-axSpA and r-axSpA in order to describe the factors associated with either disease subtype.

## Material and methods

### Patients and methods

This study is a retrospective analysis of a cohort that was constituted for doctoral degree training. The cohort was established from 1998 to 2005 and recruited at from two referral hospitals in Mexico City—the Hospital General de México and Instituto Nacional de la Nutrición Salvador de Zubirán.

The study enrolled patients who were at least 18 years old and had a diagnosis of SpA by rheumatologist diagnostic criteria. Then they were classified according to ESSG. The SpA patients were classified as axSpA and pSpA according to the presence or absence of spinal involvement. Patients with axial symptoms were classified as axSpA regardless of peripheral involvement. Patients with peripheral disease without axial symptoms were classified as pSpA. The axSpA patients were further classified as r-axSpA if they met radiographic sacroiliitis in SIJ X-ray by mNY criteria, or nr-axSpA (diagnosis made by the local rheumatologist) if they did not have sacroiliitis on sacroiliac joint X-ray. The radiographic readings were performed by an expert blinded for the clinical information and diagnosis (RB-V).

A structured and previously validated questionnaire (OMERACT IV [Outcome Measures in Rheumatology]) was administered to SpA patients, this questionnaire containing more than 100 variables, including demographic, clinical, laboratory, immunogenetic, radiologic data, disease activity, and functional limitation.^11^. Patients underwent peripheral and axial joints and entheses assessments, spinal mobility measures (modified Schober’s test, chest expansion, and occiput-to-wall distance), disease activity [painful enthesis counts (Mander), painful and inflamed joints counts, erythrocyte sedimentation rate (ESR), and C reactive protein (CRP), Bath Ankylosing Disease Activity Index (BASDAI)], functional assessment [Bath Ankylosing Spondylitis Functional Index (BASFI), Dougados functional index, and Bath Ankylosing Spondylitis Patient Global Score (BAS-G)], HLA-B27 and other HLA alleles typing, and radiographic studies of the pelvis to evaluate sacroiliac joints.

### Statistical analysis

The sample calculation included all patients evaluated in the two reference centers from 1998 to 2005, without using probabilistic methods. Nominal and ordinal variables were grouped as categorical variables and presented in absolute and/or relative frequencies, discrete and continuous variables through central tendency and dispersion measures. The Mann–Whitney U test and Chi-square with Fisher's correction were used for comparisons. Logistic regression calculated odds ratios and 95% confidence intervals. A p-value < 0.05 was considered statistical significance. SPSS (Statistical Package for the Social Sciences, IBM) version 26.0^®^ was used for statistical analysis.

### Statement of human and animal rights

All patients in the cohort signed informed consent. The confidentiality of the information was maintained, and the principles of the Helsinki Declaration were followed.

### Ethical aspects

The research protocol has received ethical and methodological approval from the research departments of the Hospital General de Mexico Eduardo Igea and the Hospital Salvador de Zubirán. It constitutes a part of the mastery degree thesis of John Londono, Julio Casasola, and Huertas-Sil, as well as the doctoral degree thesis of Cesar Pacheco. Both institutions are affiliated with the Faculty of Medicine at the Universidad Nacional Autonóma de Mexico.

## Results

### Patients and baseline characteristics

A total of 148 patients were included [70 (47%) patients nr-axSpA, 55 (37%) patients r-axSpA and 23 (16%) patients (16%) pSpA]. Male sex was 70%, with a male-to-female (M: F) ratio of 2.3:1. The median age was 28 years (IQR 22–33 years). The median age of symptom onset was 20 years (IQR 14–26 years), and the median age of diagnosis was 23 years (IQR 17–29 years). The delay in diagnosis was 2 years (IQR 0–5). HLA-B27 was present in 57% of all patients (Table 1, and supplementary Table 1).

**Table 1:** Characteristics of subgroup SpA patients.

	Total SpA, N: 148	r-axSpA, n: 55 (37.2)	nr-axSpA, n: 70 (47.3)
Men	103 (69.8)	43 (78.2)^¥^	41 (58.6)^¥^
Male:female ratio	2.3:1	3.6:1	1.4:1
Age at symptom onset (yrs)	20 (14–26)	16 (12–21)^∆^	21 (17–28)^∆^
Age at diagnosis (yrs)	23 (17–29)	19 (16–26)	26 (18–33)
Delay in diagnosis (yrs)	2 (0–5)	3.5 (0–7)	1.5 (0–5)
BMI	23.1 (20.8–25.3)	21.8 (19.9–24.3)	23.5 (21.4–23.3)
HLA-B27	85 (57.4%)	38 (92.7%)^¥^	38 (54.3%)^¥^

The information is displayed in the form of n (%) for categorical variables, whereas for continuous variables, n (IQR) is used.

*HLA-B27* human leukocyte antigen allele B-27, *BMI* body mass index, *SpA* spondyloarthritis, *r-axSpA* radiographic axial spondyloarthritis,* nr-axSpA* non-radiographic axial spondyloarthritis.

^¥^P < 0.05, ^†^P < 0.001, ^∆^P < 0.0001.

### r-axSpA vs. nr-axSpA

**Figure 1:**
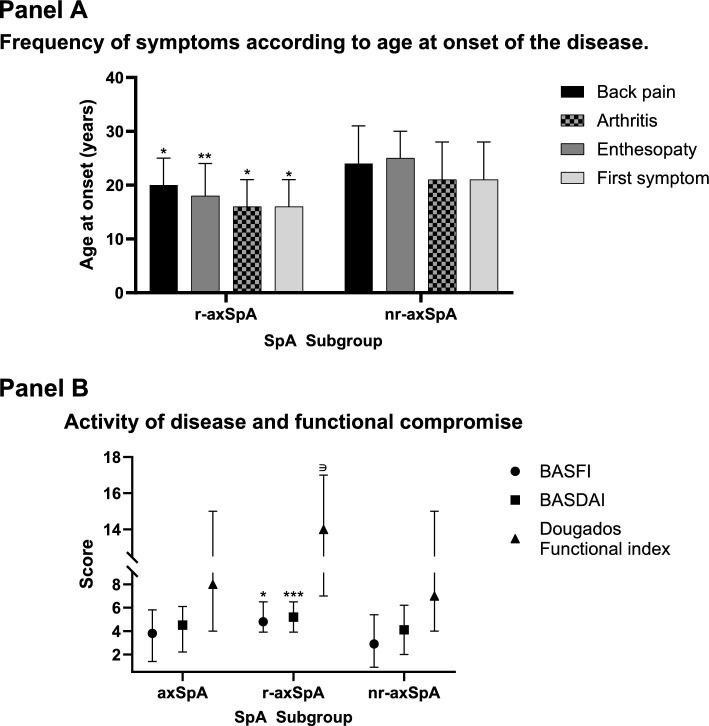
(**A**) Frequency of symptoms according to age at onset of the disease. (**B**) Activity and functional compromise of the disease. The error bar represents the upper limit. Comparison was made against nr-axSp: *P < 0.001; **P = 0.001; ***P = 0.04; ∋P = 0.01.

The female sex frequency was higher in the nr-axSpA group than r-axSpA (41.4% vs. 21.8%; P < 0.05, respectively). The frequency of HLA-B27 was lower in nr-axSpA (54.3% vs. 92.7%; P < 0.05) compared to the r-axSpA group. Patients with r-axSpA were younger at disease diagnosis (19 vs. 26 years; P < 0.0001), at first symptom onset (16 vs. 21 years; P < 0.0001), at arthritis onset (16 vs. 21 years; P < 0.05), at enthesopathy onset (18 vs. 25 years; P < 0.01), and at the age of onset of spinal pain (20 vs. 24 years; P < 0.0001) when compared to nr-axSpA, respectively (Fig. 1A).

### Clinical manifestations

Patients with nr-axSpA had a history of infection at disease onset (32.9% vs. 9.1%; P < 0.05) and less frequency of uveitis (10% vs. 25.5% P < 0.05) than patients with r-axSpA (Table 2).

**Table 2:** Symptoms according to axSpA subgroup.

Symptoms	SpA, 148 patients	r-axSpA, 55 patients (37.2)	nr-axSpA, 70 patients (47.3)
History of uveitis	24 (16.2)	14 (25.5)^¥^	7 (10)^¥^
History of infection	32 (21.6)	5 (9.1)^¥^	23 (32.9)^¥^
Fever at disease onset	56 (37.8)	19 (34.5)	29 (41.4)
Insidious onset of arthritis	27 (18.2)	9 (16.4)	14 (20)
Asymmetric arthritis	125 (84.5)	44 (80)	61 (87.1)
Additive arthritis	111 (75)	38 (69.1)	55 (78.6)
Spinal pain	99 (66.8)	47 (85.5)	52 (74.3)
Cervical pain	62 (41.9)	30 (54.5)	32 (45.7)
Thoracic spine pain	41 (27.7)	21 (38.2)	20 (28.6)
Low back pain	99 (66.8)	47 (85.5)	52 (74.3)
Anterior chest pain	43 (29.1)	20 (36.4)	23 (32.9)
Lateral chest pain	13 (8.8)	8 (14.5)	5 (7.1)
Total duration of spinal pain (month)	8 (1–27.8)	24 (10–54)^*∆*^	6 (1–18)^*∆*^
Insidious onset of spinal pain	66 (44.6)	29 (52.7)	37 (52.9)
Spinal pain with morning stiffness	85 (57.4)	32 (58.2)	53 (75.7)
Low back pain that increases with rest	82 (55.4)	28 (57.1)^¥^	54 (77.1)^¥^
Low back pain that improves with exercise	91 (61.5)	35 (71.4)	56 (80)

The information is displayed in the form of n (%) for categorical variables, whereas for continuous variables, n (IQR) is used.

*r-axSpA* radiographic axial spondyloarthritis, *nr-axSpA* non-radiographic axial spondyloarthritis, *SIJ* sacroiliac joint.

^¥^P < 0.05, ^†^P < 0.001, ^∆^P < 0.0001.

Patients with nr-axSpA had higher frequency of dactylitis (12.9% vs. 0%, P < 0.001) and less involvement in axial and peripheral skeletal entheses (P < 0.05) when compared to r-axSpA. As expected, r-axSpA patients had greater impairment in axial skeletal mobility than individuals with nr-axSpA (thoracic expansion, modified Schober test, and lateral flexion of the thoracolumbar segment of the spine (P < 0.001) (Table 3).

**Table 3:** Physical examination and clinimetry.

	Total SpA, 148	r-axSpA, 55 (37.2)	nr-axSpA, 70 (47.3)
Dactylitis on physical examination	10 (6.8)	0 (0%)^†^	9 (12.9%)^†^
Cervical spine movement limitation	30 (20.3)	24 (43.6%)^∆^	6 (8.6%)^∆^
Hip movement limitation	30 (20.3)	25 (45.5%)^∆^	4 (5.7%)^∆^
SIJ pain	46 (31.1)	22 (40%)^∆^	21 (30%)^∆^
Count of painful entheses	3 (0–9)	6 (2–12)^¥^	3 (0–9)^¥^
Axial enthesopathy count	2 (0–5)	4 (0–7)^¥^	1 (0–4)^¥^
Peripheral enthesopathy count	1 (0–4)	2 (0–5)	2 (0–3)
Thoracic expandability (cm)	4 (3–5)	3 (2–4)^∆^	4 (3.4–5)^∆^
Modified Schober test (cm)	5 (3–5.5)	3 (2–5)^∆^	5 (4–5.5)^∆^
Right lateral flexion test (cm)	4 (3–5)	3 (2–4.5)^∆^	4.5 (3.5–5)^∆^
Left lateral flexion test (cm)	4 (3–5)	3 (2–4.5)^∆^	4.5 (3.5–5)^∆^

The information is displayed in the form of n (%) for categorical variables, whereas for continuous variables, n** (**IQR**)** is used.

*r-axSpA* radiographic axial spondyloarthritis, *nr-axSpA* non-radiographic axial spondyloarthritis, *SIJ* sacroiliac joint.

^¥^P < 0.05, ^†^P < 0.001, ^∆^P < 0.0001.

### Disease activity, function, and clinical measures

Functional capacity measured by BASFI (4.8 vs. 2.9; P < 0.0001), Dougados functional index (14% vs. 7; P < 0.05), and disease activity measured by BASDAI (5.2 vs. 4.1; P < 0,001) were higher in patients with r-axSpA, when compared to nr-axSpA, respectively. In other measurements such as the BAS-G, no difference was found between the two groups. Spinal pain intensity was equally severe in both disease subtypes (Fig. 1B).

Factors associated with either disease subgroup of SpA: the factors that were found to have a higher association with r-axSpA were: a history of uveitis (OR 14, 95% CI; 2.3–85), the presence of HLA-B27 (7.97, 95% CI; 3.0–122), male sex (6.16, 95% CI; 1.5–26), a higher count of axial enthesopathy (1.17 95% CI; 1.03–1.3) and an earlier age at symptom onset (1.04 95% CI; 1–1.1) (Table 4).

**Table 4:** Factors associated with axSpA.

Feature	OR	95% CI	P value
HLA-B27	7.97	(1.35–46.7)	0.02
Sex	6.16	(1.47–25.7)	0.02
Axial enthesopathy count	1.17	(1.02–1.33)	0.004
History of uveitis	14.09	(2.3–85.2)	0.04
History of infection	0.194	(0.04–0.9)	0.04
Age at symptoms onset	1.04	(1–1.1)	0.05

*HLA-B27 *human leukocyte antigen B27, *OR* odds ratio.

## Discussion

This cohort included patients who sequentially consulted at two outpatient clinics of two specialized referral hospitals for rheumatic diseases in Mexico City, institutions with a reference population of approximately 22 million inhabitants. It is a cohort of young SpA patients with short diagnosis delay, axial presentation predominance, and a proportion of HLA-B27 allele of 57%. The symptoms more frequent in SpA patients were asymmetric arthritis, back pain, and additive arthritis. The most frequent inflammatory back pain characteristic was pain that improves with exercise. According to the results of the physical examination, sacroiliitis was observed as the most frequent sign.

The nr-axSpA presentation was the most frequent form of axSpA, with a lower proportion of male sex and frequency of HLA-B27 than r-axSpA. The nr-axSpA had late symptom onset, a highest age at diagnosis, and less delay in diagnosis than r-axSpA. The nr-axSpA had more history of infection, fever at disease onset, insidious, asymmetric, and additive arthritis, less spinal pain, and duration of spinal pain. nr-axSpA had more dactylitis, less spinal movement limitation, sacroiliac joint pain, BASDAI, and BASFI than r-axSpA.

The diagnosis of nr-axSpA was 1.4 times higher than the r-axSpA. Nevertheless, nr-axSpA/r-axSpA proportion varies globally depending on the studied region and population ^12–14^.

An association between age at disease onset and the SpA subtype was identified in our cohort. Patients with r-axSpA had an earlier disease onset, around 16 years old, and experienced a more prolonged cumulative inflammatory spinal pain during follow-up than nr-axSpA. Published evidence about the relationship between age and SpA subgroup has been contradictory; studies such as the one by Poddubnyy D et al.,^14^ that included Latin-American patients, Rudwaleit et al.^15^, and Kiltz et al.,^16^ in a German population, Kishimoto et al.,^17^ using data from ASAS-COMOSPA study, and Garcia Salinas et al.^18^ in Argentinian cohort of patients with chronic low back pain did not find differences between age and SpA subgroup. A multinational study with 914 African, Asian, and European patients, which included only 26 (2.8% of the total) Latin American patients, also found no differences^19^. We previously reported in this cohort that patients who progressed to AS tended to start the disease before age 16 compared to uSpA forms. Low-grade sacroiliitis was the main factor associated with the progression to AS^19^. In addition, Ciurea et al.^20^ observed a difference in the age of disease onset in a multinational study with a population of primarily European descent: 24.2 (IQR 19.7–30.5) for r-axSpA vs. 27.7 (IQR 22.5–35.1) for nr-axSpA, P < 0.001^20^.

Patients with nr-axSpA did not differ in sex; their male-to-female ratio was 1.4:1, as opposed to the r-axSpA group, where it was 3.6:1, p < 0.05. Similar results were reported in a systematic review that included 8 studies with 2236 patients, mainly from European databases. 70% male predominance for r-axSpA and 46.8% for nr-axSpA^21^. In PROOF study that included Latin-American patients found higher proportion of male sex in r-axSpA group, while the sex distribution was similar in the nr-axSpA group. In another study published by Zhixiu Li, et al. in a Swiss population, there was male predominance in the r-axSpA group (3:1). In contrast, in the nr-axSpA group, the distribution was equal (1:1)^22^. Kiltz et al., in a German population, also found a lower proportion of men in the nr-AxSpA group, and there was even a predominance of the female sex (68.2%)^16^. Lopez-Medina et al., also found the same findings in a systematic literature review with 9423 patients^23^. This increased risk of radiographic progression in males has been previously described^24^.

The frequency of HLA-B27 in Latin American patients with SpA is variable; in Mexican patients, it is 37%^6^ and 40.9% in Colombian patients^25^, with lower frequency than those reported in other continents: 74% to 84%^15,20,26^. Our results shows that HLA-B27 was present in 57.4% of the SpA patients. The frequency of HLA-B27 allele in r-axSpA was 92%, which is comparable with data from Caucasian population, where the frequency of HLA-B27 in patients with r-axSpA was 83%^27^. For the nr-axSpA subgroup, frequency of HLA-B27 was 54% (P = 0.01). This difference in HLA-B27 frequency between nr-axSpA and r-axSpA has been reported in other registries, such as in the German population^15^ and the SPACE cohort^28^. Some studies reported variable frequencies of the HLA-B27 allele in r-axSpA and nr-axSpA: GESPIC 2009, 73.1 vs. 74.7%^15^; Herne et al. 2012, 89.1 vs. 86.4%^16^; SCQM 2014, 82.5 vs. 78.5%^29^; DESIR cohort, 74 vs. 53.8%^30^ and ASAS-COMOSPA, 92.4 vs. 90.6%, respectively^17^. In another cohort from Latin-America, García Salinas et al.^18^ found no differences in the frequency of HLA-B27 between r-axSpA (47%) and nr-AxSpA (34%), P = 0.21. This variability in the association could be explained by the presence of alleles related to SpA other than HLA-B27: HLA-B39 in Japan population^31^ and France^32^, HLA-B14 in Sub-Sahara, HLA-Bw62 (belonging to the B15 allele) in United Kingdom^33^, and HLA-B15 in Mexico, Colombia^34^, Tunisia^35^ and Belgium^33^.

Concerning extra-articular manifestations, we found a higher incidence of uveitis in the r-axSpA group than in nr-axSpA. These results are like those reported in other studies. For example, De Winter et al. compared peripheral and extra-articular manifestations between patients with r-axSpA and those with nr-axSpA;^22^ only uveitis was greater in the r-axSpA group than in the nr-axSpA group (23% vs. 15.9%)^21^. In other studies, such as those by Kiltz et al.^16^, Rudwaleit et al.^15^, Ciurea et al.^20^, and DESIR cohort^30^, this difference seems to be less pronounced.

Our investigation showed no difference between patients with r-axSpA and those with nr-axSpA regarding the frequency of psoriasis, enthesitis, or arthritis. This lack of correlation is comparable to findings published by Garcia Salinas et al.^18^ Ruwdaleit^15^, Baraliakos^26^, and van den Berg^28^ reported. However, this study found that dactylitis was more common in the nr-axSpA group. Similar results were observed by Ciruea et al., 5.9% for r-axSpA and 11.3% for nr-axSpA^20^. On the contrary, there were no differences in the prevalence of dactylitis between r-axSpA and nr-axSpA in the ASAS-COMOSPA^17^ and DESIR^30^ cohorts. Our results show that none of the patients with r-axSpA had a history of dactylitis.

According to the findings of the current study, axial enthesitis and the total number of painful entheses are associated with r-axSpA. In contrast, comparable numbers of painful peripheral entheses were detected in both groups. In contrast, in the DESIR cohort, Lopez-Medina et al., found higher peripheral entheses (60.1 vs. 47.6%) and heel enthesitis (48.9% vs. 37.1%) in nr-axSpA than r-axSpA^30^. Winter et al.^21^, also reported a contrasting result; they identified that the presence of enthesitis favored the diagnosis of nr-axSpA (28.8% for r-axSpA and 35.4% for nr-axSpA, P 0.003)^21^. However, this association between enthesitis and a specific SpA subtype was not observed in most of the previously cited research; Kiltz^16^, Baraliakos^16^, van den Berg^28^, and ASAS-COMOSPA cohort^17^. It is interesting that in most of the studies referenced, there is no discrimination between axial and peripheral enthesitis, probably because the Mander score was not used to count painful entheses. In previous reports, both disease activity (BASDAI, low back pain score, and global pain score), and functional involvement (BASFI), are comparable between the r-axSpA and nr-axSpA groups^16,36^. In our study, we found that patients with r-axSpA had more disease activity (measured by BASDAI) and functional involvement (measured by BASFI and Dougados functional index) than patients with nr-axSpA. In other studies, in terms of BASDAI, Garcia Salinas et al.^18^, Rudwaleit et al., did not find significant differences^15^, neither the study by Poddubnyy et al.^36^, or Kiltz et al.^16^. In the DESIR cohort^30^, the nr-axSpA group had a significantly higher BASDAI (mean 46.2 vs. 40, P 0.001), but there were no differences in BASFI between the two groups. In ASAS-COMOSPA study, Kishimoto et al., found no differences in BASDAI or BASFI between two groups. In multicenter research that included Latin American patients, Burgos-Vargas et al.^19^ reported that patients with r-axSpA had a higher BASDAI, like our findings. Our findings revealed that there was no difference in axial pain severity across groups.

In this retrospective cohort, patients were biologic naïve, allowing a better description of the natural history of the disease. Patients were initially classified according to the ESSG criteria. As one of our limitations, we found that some patients could not be reclassified according to the ASAS criteria, mainly because ASAS axial criteria require the presence of the HLA-B27 allele or sacroiliitis by imaging. For example, a patient with inflammatory low back pain and enthesitis but with alleles other than HLA-B27 and without sacroiliitis by imaging cannot be classified as axial SpA according to ASAS. We previously describe the presence of alleles other than HLA-B27 that are also associated with the disease^34^. Another limitation of our study is the years in which the data were collected. This was before the ASAS classification criteria; due to the temporality of the study all patients did not have access to MRI, which indicates another important limitation, this forced us to use only the rheumatologist's criteria and conventional SIJ radiography (mNY criteria) to classify patients as nr-axSpA and could generate difficulties when comparing cohorts based on the ASAS classification. This historical study is limited to the conditions during data collection and may not represent current axSpA patients in Mexico. The small number of Mexican patients with SpA does not allow the generalization of the results to the entire Mexican population, so there is the possibility that the clinical characteristics of the disease may vary between regions and our results.

## Conclusion

This study provides information on factors that differentiate nr-axSpA from r-axSpA in a Mexican cohort. Most patients with r-axSpA disease were male, had an earlier disease onset, were HLA-B27 positive, had a greater frequency of uveitis, fewer episodes of dactylitis, more axial enthesitis, and higher disease activity. In contrast, the lack of these characteristics is associated with the absence of radiographic axial involvement (nr-axSpA).

### Supplementary Information

Supplementary Table 1.

## Data Availability

The authors have full control of all primary data and agree to allow the journal to review their data if requested.
