# Supplementary material for: Differences between radiographic and non-radiographic axial spondyloarthritis patients in a Mexican cohort
Source: Sci Rep. 2024 May 6;14:10342. doi: 10.1038/s41598-024-61001-w (PMC11074148; doi:10.1038/s41598-024-61001-w)
Supplement: Supplementary file 1 — Supplementary Table 1. [file 41598_2024_61001_MOESM1_ESM.docx]

| *Supplementary Table 1: Antecedents of SpA patients according to axSpA subgroups* | | | |
| --- | --- | --- | --- |
|  | r-axSpA  55 patients (37.2) | nr-axSpA  70 patients (47.3) | pSpA  23 patients (15.5) |
| *History of dactylitis* | 15 (27.3) | 25 (35.7) | 6 (26.1) |
| *History of enthesopathy* | 37 (67.3) | 45 (64.3) | 11 (47.8) |
| *History of psoriasis* | 1 (1.8) | 1 (1.4) | 0 |
| *History of IBD* | 3 (5.5) | 5 (7.1) | 1 (4.3) |
| *History of uveitis* | 14 (25.5) | 7 (10) | 3 (13) |
| *Familiar with SpA* | 16 (29.1) | 22 (31.4) | 3 (13) |

r-axSpA: radiographic axial spondyloarthritis; nr-axSpA: non-radiographic axial spondyloarthritis; pSpA: Peripherical Spondyloarthritis.

The information is displayed in the form of n **(**%**)** for categorical variables, whereas for continuous variables, n **(**IQR**)** is used.
